# Cardiac, Metabolic and Molecular Profiles of Sedentary Rats in the Initial Moment of Obesity

**DOI:** 10.5935/abc.20170151

**Published:** 2017-11

**Authors:** Bruno Barcellos Jacobsen, Ana Paula Lima Leopoldo, Jóctan Pimentel Cordeiro, Dijon Henrique Salomé de Campos, André Ferreira do Nascimento, Mário Mateus Sugizaki, Antônio Carlos Cicogna, Carlos Roberto Padovani, André Soares Leopoldo

**Affiliations:** 1 Universidade Federal do Espírito Santo (UFES), Vitória, ES - Brazil; 2 Departamento de Clínica Médica - Faculdade de Medicina - UNESP, Botucatu, SP - Brazil; 3 Universidade Federal de Mato Grosso (UFMT), SINOP, MT - Brazil

**Keywords:** Rats, Obesity, Diet, Hight-Fat, Cardiac function, Calcium, Adiposity

## Abstract

**Background:**

Different types of high-fat and/or high-energy diets have been used to induce obesity in rodents. However, few studies have reported on the effects observed at the initial stage of obesity induced by high-fat feeding on cardiac functional and structural remodelling.

**Objective:**

To characterize the initial moment of obesity and investigate both metabolic and cardiac parameters. In addition, the role of Ca^2+^ handling in short-term exposure to obesity was verified.

**Methods:**

Thirty-day-old male Wistar rats were randomized into two groups (n = 19 each): control (C; standard diet) and high-fat diet (HF, unsaturated high-fat diet). The initial moment of obesity was defined by weekly measurement of body weight (BW) complemented by adiposity index (AI). Cardiac remodelling was assessed by morphological, histological, echocardiographic and papillary muscle analysis. Ca^2+^ handling proteins were determined by Western Blot.

**Results:**

The initial moment of obesity occurred at the 3^rd^ week. Compared with C rats, the HF rats had higher final BW (4%), body fat (20%), AI (14.5%), insulin levels (39.7%), leptin (62.4%) and low-density lipoprotein cholesterol (15.5%) but did not exhibit alterations in systolic blood pressure. Echocardiographic evaluation did not show alterations in cardiac parameters. In the HF group, muscles were observed to increase their +dT/dt (C: 52.6 ± 9.0 g/mm^2^/s and HF: 68.0 ± 17.0 g/mm^2^/s; p < 0.05). In addition, there was no changes in the cardiac expression of Ca^2+^ handling proteins.

**Conclusion:**

The initial moment of obesity promotes alterations to hormonal and lipid profiles without cardiac damage or changes in Ca^2+^ handling.

## Introduction

Obesity is considered a major syndrome of the XXI century and has reached epidemic proportions worldwide in recent decades.^1,2^ According to the World Health Organization, the number of overweight individuals has reached over a billion people, and more than 30% of this population is obese.^3^ Obesity is a complex disease, and while some authors have suggested that genetic factors contribute its development,^4^ most research emphasizes that major causes of obesity are the so-called exogenous factors, especially the consumption of highly available, palatable food, and lack of exercise.^2,5^

A number of different types of high-fat and/or high-energy diets have been used to induce obesity and mimic human metabolic syndrome in rodents.^6-11^ However, few studies have investigated the initial stage of obesity induced by a high-fat diet. Researchers have observed the initial moment of obesity in animals fed a high-fat diet after 4 weeks of treatment.^12-15^ However, molecular and cardiac parameters in this initial stage were not presented.

Studies have shown that excess body fat leads to several cardiovascular abnormalities that correlate with the duration and intensity of obesity in humans and in animal models.^16-20^ Thus, it becomes necessary to identify the duration and intensity of damage in the early period of the disease. Furthermore, it is important to verify the mechanisms involved in this process, since studies have shown that abnormalities in Ca^2+^ handling may be responsible for the development of cardiac dysfunction in obesity models induced by a high-fat diet.

Due to the lack of studies, our purpose was to characterize the initial moment of obesity and investigate both the metabolic and cardiac parameters in obese rats. In addition, the role of Ca^2+^ handling in short-term exposure to obesity was evaluated.

## Methods

### Animal Care

All experiments and procedures were performed in accordance with the *Guide for the Care and Use of Laboratory Animals* published by the U.S. National Institutes of Health and approved by the Botucatu Medical School Ethics Committee (UNESP, Botucatu, SP, Brazil) (approval numberFMB-PE-5/2009).

Thirty-day-old male *Wista*r rats were distributed into two groups: control (C, n = 19) and high-fat diet (HF, n = 19). Group C was fed a standard diet containing 12.3% of its energy from fat, 57.9% from carbohydrate, and 29.8% from protein.The HF animals were fed four high-fat diets (RC Focus 2413, 2414, 2415, and 2416) that differed in their flavouring but not in their micro- and macronutrients. The high-fat diets contained 49.2% of their energy from fat, 28.9% from carbohydrates, and 21.9% from protein as previously described.^17^ All rats were housed in individual cages in an environmentally controlled, clean-air room at 23 ± 3°C with a 12-h light/dark cycle (lights on at 6am) and 60 ± 5% relative humidity. After starting the experimental protocol, food consumption (FC), energy intake (EI), feed efficiency (FE), and body weight (BW) were recorded weekly. EI was calculated as follows: EI = average weekly EI multiplied by the caloric value of each diet (C or HF). FE (%) is the ability to convert EI to BW and was determined as the mean BW gain (g)/total calorie intake (kcal) x100.

### Characterization of the Initial Moment of Obesity

After starting the experimental protocol, BW was recorded once a weekto characterize the initial moment of obesity. When C and HF groups presented a significant difference in BW, the animals were anesthetized by ketamine injection (50 mg/kg) and xylazine (0.5 mg/kg) intraperitoneal (IP) injection, decapitated, and thoracotomized, and the fat pads were dissected and weighed. The initial moment of obesity was defined by BW (g) measurements that were recorded weekly and complemented by *post-mortem* adiposity index (AI) using the following formula: AI = [body fat (BF)/ BW] x 100. BF (g) was measured from the sum of the individual fat pad weights: epididymal fat + retroperitoneal fat + visceral fat.

### Systolic blood pressure

The systolic blood pressure (SBP) of the tail was measured one week before euthanasia with a tail plethysmograph. The animals were warmed in a wooden box at 40°C with heat generated for four minutes to cause vasodilation of the tail artery, and the animals were then transferred to an iron cylindrical support. A sensor was placed in the proximal region of the tail and coupled to an electro-sphygmomanometer (NarcoBioSystem, International Biomedical Inc, TX, USA). The electro-sphygmomanometer was attached to a computer,and SBP was measured using the Biopac software (Biopac Systems Inc., CA, USA).

### Insulin tolerance test (ITT)

Blood samples were drawn from the tip of the tail at basal condition and after the intraperitoneal administration of regular insulin (Novolin^2^ R, Novo Nordisk, Bagsvaerd, Denmark) at a dose of 1.5 IU/kg body weight.^21^ Blood glucose was then collected at 0 (basal), 5, 10, 15, 20, 25 and 30 min. Glucose levels were determined using a handheld glucometer (Accu-ChekAdvantage; Roche Diagnostics Co., Indianapolis, USA). Insulin resistance was determined from the area under the curve for glucose (AUC; 0-30 minutes).

### Echocardiographic evaluation

One week before euthanasia, echocardiographic evaluation was performed using a commercially available echocardiograph (Philips HDI-5000), and left ventricular (LV) structural variables were evaluated as previously described.^22^

### Metabolic and hormonal profile

Blood samples were collected, and the serum was separated by centrifugation at 3,000 × g for 15 minutes at 4°C. Glucose, total cholesterol (T-Chol), high-density lipoprotein cholesterol (HDL), low-density lipoprotein cholesterol (LDL), and hormones (insulin and leptin) were analysed. Glucose, TChol, HDL and LDL were measured using an automatic enzymatic analysis system (Biochemical analyzer BS-200, Mindray, China). The leptin and insulin levels were determined by enzyme-linked immunosorbent assay methodology using commercial kits (Linco Research Inc., St. Louis, MO, USA).

### Morphological and histological analysis

After the initial moment of obesity, the heart weight (HW), HW/final body weight (FBW) ratio, papillary muscle cross-sectional area (CSA) and collagen fraction (n = 14; each group) were recorded.

### Papillary Muscle Function

Papillary muscles isolated from the LV were evaluated as previously described.^17^ The papillary muscles were evaluated under the baseline condition of 2.5 mM Ca^2+^, post-rest contraction (PRC) and after elevation of extracellular Ca^2+^ concentration. PRC was studied at an extracellular Ca^2+^ concentration of 0.5 mM, in which the stimulus was paused for 10, 30, and 60 s before restarting the stimulation. Inotropic responses were recorded 5 min after the addition of each dose of extracellular Ca^2+^ (0.5, 1.0, 1.5, 2.0, and 2.5 mM) to the bathing solution.

### Western Blot analysis

LV tissue (C; n = 6; HF; n = 6) was analysed by Western Blot to quantify the L-type Ca^2+^ channel, SERCA2A: Sarcoplasmic reticulum Ca^2+^ ATPase (SERCA2a) and phospholamban (PLB). expression as previously described.^20^ Specific antibodies were obtained against SERCA2 ATPase (ABR, Affinity BioReagents, CO, USA; MA3-910, 1:2,500), PLB (ABR, Affinity BioReagents, CO, USA;MA3-922, 1:500) and L-type Ca^2+^ channel alpha 1C (Sigma-Aldrich, St. Louis, MO;C4980, 1:200). Binding of the primary antibody was detected with peroxidase-conjugated secondary antibodies (rabbit or mouse, depending on the protein). Quantification of blots was performed by Scion Image software (Scion based on NIH image). Targeted bands were normalized to the expression of b-actin by using an antibody (SC81178; 1:1000) obtained from Santa Cruz Biotechnology (CA, USA).

### Statistical analysis

The statistical analysis was performed using the Sigma Stat 3.5 software (SYSTAT Software Inc., San Jose, CA, USA). The distribution of the variables was assessed by using the Kolmogorov-Smirnov test for normality, and the results were reported as means ± standard deviation (SD). Comparisons between groups were performed using Student'st-test for independent samples and a repeated-measures two-way analysis of variance (ANOVA) when appropriate. The level of significance considered was 5%.

The sample size (n) was estimated using the equation: n = [(Z1-α/2 + Z1-β) x r/Δ)2, where n is the sample size, Z is the z score, a is the two-sided significance level (0.05; type I error), b is the statistical power (80%; type II error), r is the SD and Δ is the minimal difference between groups. ^23^ The sample size needed to detect a significant between groups is 10 rats per group; however, we decided to use 19 animals per group for most of the analyses.

## Results

BW was similar in the first two weeks of treatment in both groups C and HF (*data not shown*); however, during the third week, BW was greater in group HF than in group C. This moment was characterized as the initial moment of obesity.

Table 1 shows the general characteristics, comorbidities and hormone results from C and HF rats after characterizing the initial moment of obesity (3 weeks). The FBW and weight gain were both higher in HF than in C. The high-fat diet promoted a substantial elevation of epididymal and visceral fat pad weight and BF. However, initial BW and retroperitoneal fat pad weight did not differ between the groups. AI was higher in HF animals (14.5%) compared to C animals. Compared with C group, HF had a lower FC, a greater FE, but a similar EI. Glucose, T-Chol and HDL levels, and SBP were similar between the groups. However, the AUC for glucose obtained in the insulin tolerance test, and LDL, insulin and leptin levels were significantly higher in HF compared to C.

**Table 1:** General characteristics, comorbidities and hormones

Variables	Groups	
	C (n = 19)	HF (n = 19)
IBW, g	148 ± 12	147 ± 12
FBW, g	290 ± 18	302 ± 22*
WG, g	142 ± 10	155 ± 10*
Epididymal, g	4.6 ± 0.8	5.6 ± 1.2*
Retroperitoneal, g	5.4 ± 1.5	6.4 ± 1.7
Visceral, g	4.1 ± 1.0	4.9 ± 0.9*
BF, g	14.1 ± 3.0	16.9 ± 3.2*
AI, %	4.9 ± 1.0	5.6 ± 0.9*
Glucose, mg/dL	182 ± 27	181 ± 21
AUC, mg/dL/min	2129 ± 193	230 8 ± 218*
T-Chol, mg/dL	63.2 ± 10.4	68.3 ± 6.1
HDL, mg/dL	49.2 ± 7.7	52.9 ± 4.7
LDL, mg/dL	9.0 ± 1.7	10.4 ± 2.4*
SBP, mmHg	127 ± 8	131 ± 14
Insulin, ng/mL	0.83 ± 0.16	1.16 ± 0.28*
Leptin, ng/mL	2.34 ± 0.57	3.80 ± 1.26*

Values are means ± SD; control (C) and high-fat diet (HF) groups; n: number; IBW: initial body weight; FBW: final body weight; WG: weight gain; BF: body fat; AI: adiposity index; AUC: area under the curve for glucose determined in insulin tolerance test (ITT); T-Chol: total cholesterol; HDL: high density lipoprotein cholesterol; LDL: low density lipoprotein cholesterol; SBP: systolic blood pressure;

* p < 0.05 vs. C. Student's t-test for independent samples.

**Figure 1:**
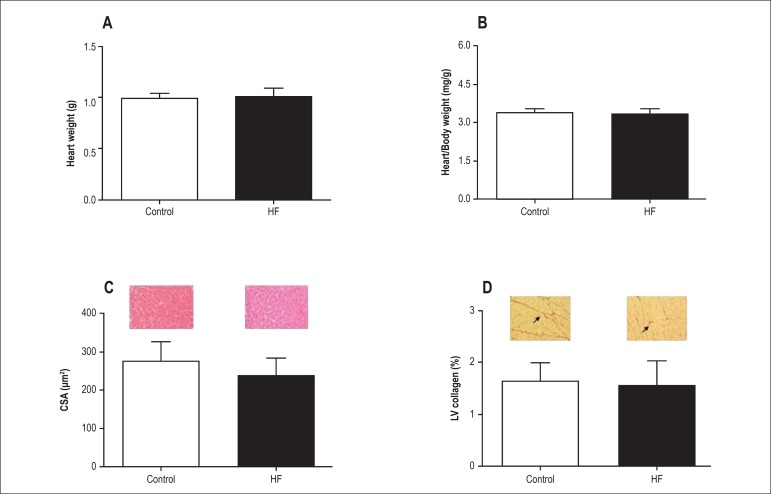
Morphological analysis of control (C) versus high-fat diet (HF) rats. A: Heart weight. B: Heart/final body weight ratio. C: Myocyte cross-sectional area (40× magnification lens); representative haematoxylin and eosin-stained left ventricular cross-sections from C and HF rats. D: Interstitial collagen volume fraction of myocardium (20× magnification lens) from C and HF rats; representative picrosirius red-stained left ventricular sections from C and HF rats. Arrows represent the interstitial collagen volume fraction of C and HF. Data presented as the mean ± SD. Student’s t test was used for independent samples. There are no differences between groups

The morphological and histological analyses are presented in Figure 1. Heart weight (Figure 1A), heart/FBW ratio (Figure 1B), myocyte CSA (Figure 1C), and LV collagen fraction (Figure 1D) were similar between the groups.

**Figure 2:**
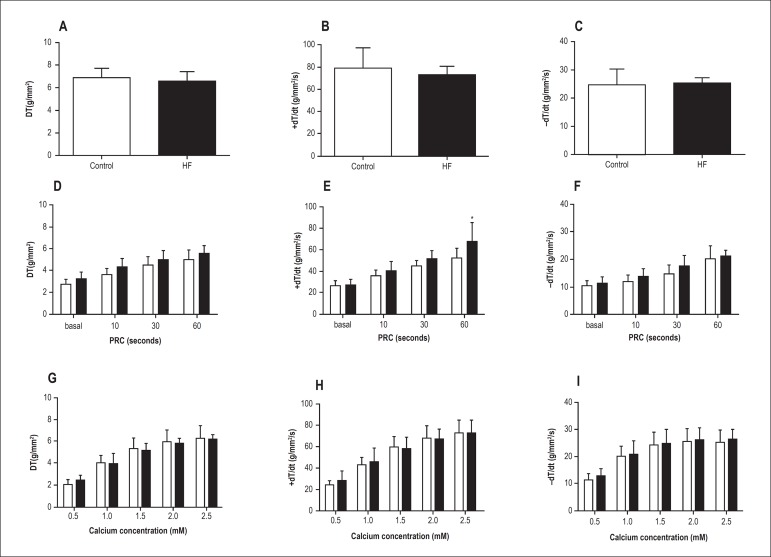
Basal condition (A, B and C), post-rest contraction (D, E and F) and effects of increasing extracellular Ca^2+^ concentration (G, H and I ) in papillary muscles of control (C) and high-fat diet (HF) rats (white bars = C; black bars = HF; n = 19 in each condition). Maximum developed tension normalized percross-sectional area (DT) [g/mm^2^], negative (-dT/dt[g/mm^2^/s]) and positive (+dT/dt[g/mm^2^/s]) tension derivatives normalized per cross-sectional area. Data presented as the means ± SD. *p < 0.05 versus C. Student’s t-test for independent samples (A, B and C) and repeated-measures two-way ANOVA (D, E, F, G, H and I); Student-Newman-Keuls post-hoc test

**Figure 3:**
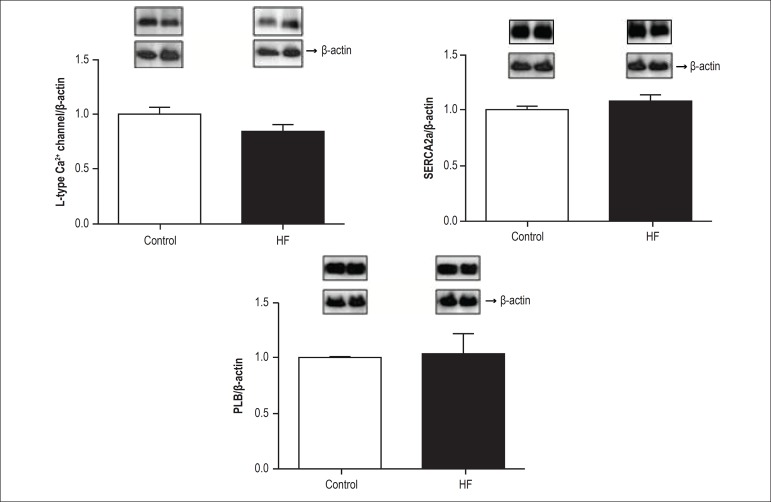
Cardiac protein expression by Western Blot. The data are means ± SD (n = 6 in each group); control (C) and high-fat diet (HF); A: L-type Ca^2+^ channel; B: Sarcoplasmic reticulum Ca^2+^ ATPase (SERCA2a) and C: phospholamban (PLB). Student’s t-test for independent samples

Echocardiographic evaluation showed that the HF rats did not show a difference in heart rate (HR), left ventricular end-diastolic dimension (LVDD), left ventricular end-systolic dimension (LVSD), posterior wall thickness in diastole (PWTd), relative wall thickness (RWT), left atrium (LA), left ventricular mass (LVM), fractional shortening (FS) endocardial, FS midwall, posterior wall shortening velocity (PWSV), earlydiastolic mitral inflow (E-wave), latediastolic mitral inflow (A-wave), early-to-late diastolic mitral inflow ratio (mitral E/A), E-wave deceleration time (EDT) or isovolumetric relaxation time (IVRT) compared to C rats (Table 2). However, the HF group presented higher aortic diameter (AO) in relation to C. After 3 weeks, the treatment did not promote contractile dysfunction in basal condition or after Ca^2+^ stimulation (Figure 2). However, the results demonstrated that during manoeuvre PRC, an increase in contractile phase was observed after 60 seconds in group HF compared to C, as visualized by positive tension derivative normalized per CSA (+dT/dt) (C: 52.6 ± 9.0 g/mm^2^/s and HF: 68.0 ± 17.0 g/mm^2^/s; p < 0.05) (Figure 2E). Figure 3(A-C) shows that 3 weeks of high-fat feeding did not alter the protein levels of the L-type Ca^2+^ channel, SERCA2a or PLB.

**Table 2:** Echocardiography data

Variables	Groups	
	C (n = 10)	HF (n= 10)
HR, bpm	341 ± 40	316 ± 22
LVDD, mm	7.85 ± 0.53	7.98 ± 0.37
LVSD, mm	3.66 ± 0.63	3.70 ± 0.44
PWTd, mm	1.47 ± 0.06	1.44 ± 0.07
RWT	0.19 ± 0.02	0.18 ± 0.01
AO, mm	3.39 ± 0.14	3.53 ± 0.13*
LA, mm	5.27 ± 0.32	5.39 ± 0.31
LVM, g	0.81 ± 0.06	0.83 ± 0.10
FS endocardial, %	53.6 ± 5.7	53.6 ± 4.5
FS midwall, %	33.0 ± 3.0	33.9 ± 3.7
PWSV, mm/s	40.38 ± 5.49	38.79 ± 3.05
E-Wave (cm/s)	94.5 ± 13.6	87.4 ± 3.9
A-Wave (cm/s)	67.4 ± 18.7	60.2 ± 4.7
Mitral E/A	1.4 ± 0.4	1.5 ± 0.2
EDT, ms	42.0 ± 4.8	43.8 ± 4.9
IVRT, ms	20.4 ± 3.1	20.1 ± 3.8

Data presented as the mean ± standard deviation. C: control and HF: high-fat diet groups; n: number; HR: heart rate; LVDD: left ventricular end-diastolic dimension; LVSD: left ventricular end-systolic dimension; PWTd: posterior wall thickness in diastole; RWT: relative wall thickness; AO: aortic diameter; LA: left atrium; LVM: left ventricle mass; FS endocardial: fractional shortening; FS midwall: fractional shortening; PWSV: posterior wall shortening velocity;early (E-wave) and late (A-wave) diastolic mitral inflow; E/A: early-to-late diastolic mitral inflow ratio; EDT: E-wave deceleration time; IVRT: isovolumetric relaxation time;

* p < 0.05 versus C (control). Student's t test for independent samples.

## Discussion

The determination of the initial moment of obesity is essential to control the duration of experimental protocols because it allows one to accurately assess the influence of exposure to adiposity and, consequently, obesity.^16^ Interestingly, little information is available on this process and its cardiovascular consequences.

Some researchers have shown metabolic, cardiac and molecular characteristics only at the end of the experiment,^17,20,24^ which precludes analyses regarding the precise onset of disturbances caused by excess adipose tissue and their intensity. A question that arises from the present study is why the experimental studies have not characterized the initial moment of obesity. Thus, the major finding was that there were alterations in the metabolic and lipid profiles at the initial moment of obesity, but without accompanying cardiac damage or changes to Ca^2+^ handling proteins.

High-fat diets are generally accepted as a method of generating a valid rodent model for obesity. According to the data obtained, we developed a valid rodent model for diet-induced obesity with unsaturated fat. After 3 weeks, HF animals showed higher weight gain (16%) and body fat (20%) than C animals. The findings are in agreement with several authors.^18-20,24^ In addition, the animal model presented some features of metabolic syndrome, such as central obesity, glucose intolerance and dyslipidemia, but without alterations in SBP, thus demonstrating obesity with its comorbidities. In contrast, previous investigations have observed numerous comorbidities associated with short-term obesity, such as hypertension and diabetes.^25,26^

The initial moment of obesity caused important metabolic abnormalities, such as elevated leptin (62.5%) and insulin (40%) levels in the HF group. According to the literature, leptin is a hormone secreted by adipose tissue and has a direct relationship with the amount of BF.^27^ The relationship between leptin levels and fat shows that the amount of leptin is approximately 3 times higher than BF. Furthermore, leptin is able to directly activate nitric oxide production via L-arginine, which is dependent on endothelial integrity^27^ and may be a determining factor in the absence of hypertension. In addition, hyperinsulinemia was observed with insulin sensitivity damage, since the glucose AUC was higher in group HF than group C.

The initial moment of obesity did not cause cardiac remodelling, as visualized by histological and echocardiographic analysis. Dhanasekaran et al.^28^ reported that insulin resistance induced by obesity with associated hyperinsulinemia could promote cardiac remodelling via the growth-promoting properties of insulin or by attenuating the anti-apoptotic signalling of the phosphatidylinositol 3' -kinase/protein kinase B pathway.^29^ Studies in mice with (functional) leptin deficiency have suggested that the cardiac hypertrophy developing in states of chronic hyperleptinaemia may result from an inability to transduce anti-hypertrophic and/or cardioprotective effects of the adipokine.^29^

Nonetheless, obesity is still considered a risk factor in the development of cardiovascular disorders, despite the called "obesity paradox". A number of rodent models of obesity have been studied in terms of cardiovascular adaptations. ^12,15,24^ Diet-induced obese rats exhibit many of the hemodynamic alterations associated with human obesity, but there is no evidence to date that these animals will develop severe cardiac depression. In the current study, echocardiographic and papillary muscle analysis showed that there was no change in cardiac parameters in both groups. The absence of functional changes may be due to the short term of the exposure to HF diet.

According to the results, short-term exposure to HF diet promoted specific changes at contraction phase after the PRC manoeuvre, indicating absence of myocardial function impairment. This result could be related to Ca handling changes; however, our results show that there was no change in the levels of Ca^2+^ L-type channels, PLB or SERCA2a protein, suggesting that the kinetic properties of calcium are preserved in the onset of obesity. Furthermore, the post-translational modifications known to affect the activity of these proteins, such as phosphorylation and glycosylation, were not investigated in the present study.

### Study limitations

The study did not investigate post-translational modifications known to affect the activity of proteins, such as phosphorylation and glycosylation, which could consolidate the absence of alterations in the expression of proteins involved in the intracellular calcium handling at the initial moment of obesity.

## Conclusion

The initial moment of obesity promotes alterations to hormonal and lipid profiles without cardiac damage and changes to Ca^2+^ handling in a rat model of unsaturated high-fat diet-induced obesity. Taken together, these findings could be relevant to human pathology and enable the verification and prevention of disturbances in the early period of obesity.

## Acknowledgments

This manuscript has been proofread and edited by native English speakers with related biomedical backgrounds from the American Journal Experts.
